# Searching in Euclidean Spaces with Predictions

**DOI:** 10.1007/s00224-026-10291-w

**Published:** 2026-09-05

**Authors:** Sergio Cabello, Panos Giannopoulos

**Affiliations:** 1 Faculty of Mathematics and Physics, University of Ljubljana, Ljubljana, Slovenia; 2 Department of Computer Science, City St. George’s, University of London, London, UK; 3 Institute of Mathematics, Physics and Mechanics, Ljubljana, Slovenia

**Keywords:** Search games, Predictions, Distance, Computational geometry, Theory of computation, Computational geometry

## Abstract

We study the problem of searching for a target at some unknown location in $\mathbb {R}^d$ when additional information regarding the position of the target is available in the form of predictions. In our setting, predictions come as approximate distances to the target: for each point $p\in \mathbb {R}^d$ that the searcher visits, we obtain a value λ(p) such that $|p\boldsymbol{t}|\le \lambda (p) \le c\cdot |p\boldsymbol{t}|$, where c≥1 is a fixed constant, $\boldsymbol{t}$ is the position of the target, and $|p\boldsymbol{t}|$ is the Euclidean distance of *p* to $\boldsymbol{t}$. The cost of the search is the length of the path followed by the searcher. Our main positive result is a strategy that achieves $O(c^d)$-competitive ratio, even when the constant *c* is unknown. We also give a lower bound of roughly $(c/4)^{d-1}$ on the competitive ratio of any search strategy in $\mathbb R^d$, assuming that c≥4.

## Introduction

The problem of searching for a target positioned at some unknown location in some region is a classic search game problem that has been well-studied in both fields of Computational Geometry and Operations Research. The problem comes in many different versions, such as linear search (i.e., searching for a target on a line) [6, 7, 9, 16], searching in the plane [4, 8, 17, 23], searching in concurrent rays [4, 15, 16], and searching inside polygonal regions [27, 33]. The book by Alpern and Gal [1] provides an extensive overview of general search games, while Ghosh and Klein [18] survey search problems in planar domains.

The searcher starts from some given position, follows some path according to some strategy until the target, usually a point, is reached or detected, for some appropriate definition of “detection”. In 1-dimensional settings, e.g., an infinite line, one usually requires that the searcher passes through the target point. In the plane one usually requires that the searcher is within some distance of the target, or sees the target, if there are obstacles, or that the target lies on the segment connecting the searcher’s current and starting positions [17, 18, 29].

The cost of the search is the length of the path followed by the searcher and the objective is to find an efficient search strategy. Efficiency is usually measured by the ***competitive ratio***, which, in this setting, is the ratio of the length of the path of the searcher to the actual Euclidean distance of the starting position to the target.

In this work, we consider the problem of finding a target point $\boldsymbol{t}$ in Euclidean space $\mathbb {R}^d$ when additional information regarding the position of the target is available in the form of ***predictions***. Here, the predictions are the approximate distance to $\boldsymbol{t}$ for all points visited during the search, e.g., a value between, say, $|p\boldsymbol{t}|$ and $2|p\boldsymbol{t}|$ for each point *p* visited, where $|p\boldsymbol{t}|$ is the Euclidean distance between *p* and *t*. Such an estimate could be obtained for example in a scenario where one takes into account the strength of a signal broadcasted by the target.

Algorithms with predictions is a concept that has been introduced relatively recently; see the survey by Mitzenmacher and Vassilvitskii [31]. The general idea is that on top of the usual input data we are also given additional and possibly inaccurate (noisy) information, the prediction, that should assist the algorithm to be more effective. The improvement in performance depends on the accuracy of the prediction.

### Problem Setup

We consider the following search problem in $\mathbb R^d$. Assume that there is a fixed but unknown ***target*** point $\boldsymbol{t}\in \mathbb R^d$. Without loss of generality, we start the search at the origin, which we denote by $\boldsymbol{o}$. We want to find a curve γ that starts at $\boldsymbol{o}$ and ends at $\boldsymbol{t}$. The ***cost*** of the search is the Euclidean length of the curve γ.

As we search for the target, we have approximate information about the distance to it from each point that we have visited so far. More precisely, we assume that there is a constant c≥1 and an unknown function

1 $$\begin{aligned} \lambda :\mathbb R^d \rightarrow \mathbb R_{\ge 0} ~\text { such that }~ \forall p\in \mathbb R^d~~ |p\boldsymbol{t}|\le \lambda (p) \le c\cdot |p\boldsymbol{t}|. \end{aligned}$$

**Fig. 1:**
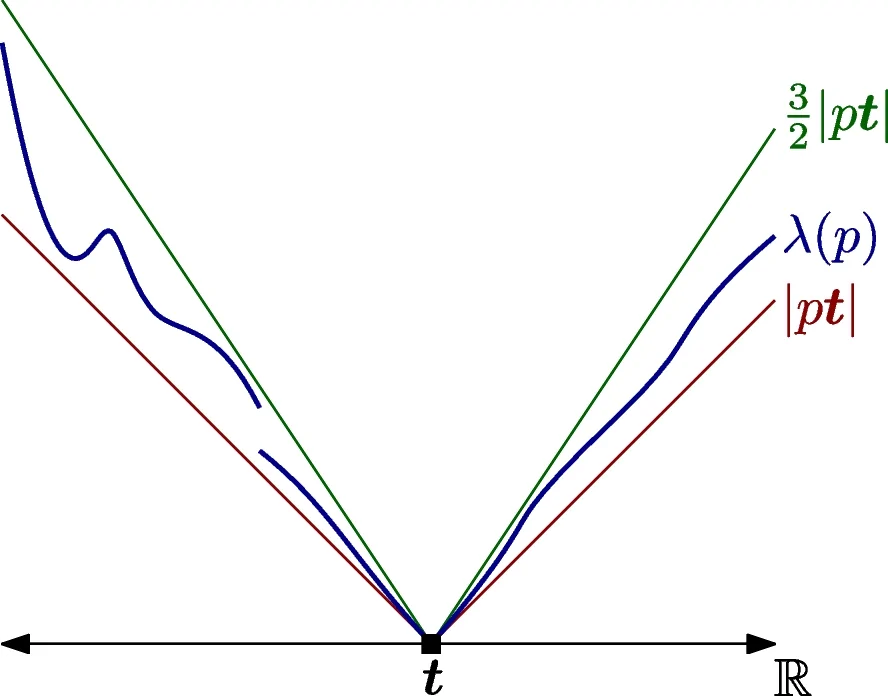
Example of *c*-prediction function λ(p) for c=3/2. In this example the function is not monotone in $|p\boldsymbol{t}|$ and it is not continuous

We refer to such a function λ as a *c****-prediction*** for the target $\boldsymbol{t}$. The constant *c* is the ***prediction factor*** of λ. See Fig. 1 for an example in d=1. Note that for c=1, the function λ gives the exact distance to the target.

For each point *p* along the search path we have traversed so far, we obtain the value λ(p), and the search strategy decides how to continue the search depending on that information. We know when we have reached the target because λ(p)=0 holds only when $p=\boldsymbol{t}$.

As it is common in search games, we are interested in the competitive ratio of the search strategy: how does the length of the search path compare to the straight-line distance from the origin to the target? To formalize this, for each target $\boldsymbol{t}\in \mathbb R^d$ and each constant c≥1, we consider the family $\Lambda (\boldsymbol{t},c)$ of *c*-predictions for $\boldsymbol{t}$, that is, functions satisfying condition (1). The family of *c*-prediction functions is $\cup _{\boldsymbol{t}\in \mathbb R^d} \Lambda (\boldsymbol{t},c)$.

A search strategy *S* is $\alpha =\alpha (S,c,d)$ ***competitive*** if for all $\boldsymbol{t}\in \mathbb R^d$ and all $\lambda \in \Lambda (\boldsymbol{t},c)$, the length of the path defined by *S* to reach $\boldsymbol{t}$ from $\boldsymbol{o}$ is at most $\alpha |\boldsymbol{o}\boldsymbol{t}|$. Note that the prediction factor *c* may be known or unknown to the search strategy.

### Our Contribution

Our main contributions are the following:

- We introduce a natural, new search problem in $\mathbb R^d$ for d≥1, under a predictions model where we have approximate information about the distance to the target.
- We show that for each dimension *d* and each constant prediction factor c≥1 there is a search strategy with competitive ratio smaller than $(5c)^{d+1}$. To achieve this, we use ε-nets from metric spaces, also known as *r*-nets, and provide a path of finite length but an infinite number of pieces. This result holds assuming that we know the prediction factor *c*. For *unknown* prediction factor, a slightly different search strategy leads to a competitive ratio smaller than $(10c)^{d+1}$. Both of these ratios can be improved to $O(c^d)$. These results are given in Sect. 4.
- We show that for c≥4, any deterministic search strategy in $\mathbb R^d$ with *c*-predictions will have a competitive ratio of at least $(c/4)^{d-1}\cdot \min \{\sqrt{\pi /d},1\}$. For this result, we construct an infinite family of *c*-predictions and use a volume argument to give a lower bound on the length of any path that can discern which *c*-prediction from the family is the actual one. The approach is motivated by the techniques used to obtain approximation algorithms for the Euclidean Traveling Salesperson with Neighbourhoods. However, in our setting we have an infinite number of neighbourhoods, one for each *c*-prediction. With a slightly worse constant, the lower bound also holds for randomized search strategies. This lower bound is shown in Sect. 5.

Additionally, we give several basic properties of the model. For example, we show that without a prediction function throughout the whole search, we cannot find the target using a path of bounded length, and having an infinite number of pieces (segments) in the search path is unavoidable. We also show that we may assume that the prediction function is continuous. We also note that when c=1, i.e., we have exact information about the distance to the target, the target can be reached with competitive ratio arbitrarily close to 1. These results are presented in Sect. 3.

To our knowledge, this is the first search problem in Euclidean spaces with d≥2 where a search point reaches a target point with constant competitive ratio; see the discussion below about related work. For our upper bounds we use bounds on the cardinality of ε-nets. For the sake of simplicity in the calculations, we use suboptimal but simple-to-parse estimates in the simplifications and the cardinality of ε-nets. Similarly, in our lower bounds we also use volume estimates that are easier to manipulate. In any case, our upper and lower bounds for the best competitive ratio are still far apart.

Our model, as presented, is continuous and has the advantage of being scale-free, a desired mathematical property. While the searcher may receive predictions at any point it wishes along the search path, in our strategies the searcher receives predictions only at the endpoints of the straight-line segments that comprise the path. Hence, only an enumerable set of predictions is used.

There are several natural *variants* of the model, and some of them may be of interest in more practical scenarios. A first variant is to assume that the target has integer coordinates or that the target can be detected when the searcher is within a given distance from it. This model is not scale-free anymore. Our search strategies can be easily modified to have a *finite* number of steps in this setting. The lower bound holds in this setting too, albeit with a slightly worse constant. A second variant is to assume that information about the distance is given only at regular intervals; to interpret this setting it is more convenient to assume that the searcher moves at unit speed and that the estimated distance to the target is available to the searcher at regular time intervals. In this case, it is meaningful to define the competitive ratio by comparing the travel times to reach the target. It follows from our results that in this setting no bounded competitive ratio is possible. If we mix the two variants we have mentioned, then simple modifications of our search strategies achieve bounded competitive ratio. Another variant is to allow for underestimates of the actual distance (in addition to the default overestimates). Our positive and negative results also hold in this setting, with slightly different constants. These variants are discussed along the text, mostly in Sect. 4.1.

### Related Work

For *linear* search, when the exact distance to the target is known, one can easily find the target by walking at most three times this distance. When the distance to the target is unknown, Beck and Newman [7] and later Baeza-Yates at al. [4] showed that a simple deterministic doubling strategy has competitive ratio 9; here, a lower bound on the distance to the target is assumed, otherwise there is no search strategy with constant competitive ratio. There are also other similar strategies with the same competitive ratio [10, 18]. Moreover, various approaches [4, 7, 22, 28] show that 9 is the best possible deterministic competitive ratio for the problem. Finally, Gal [17] and later Kao et al. [25] gave an optimal randomized strategy with competitive ratio of approximately 4.5911.

Gal [15, 16] introduced the problem of searching for a target on multiple *rays* that are concurrent at the starting position and gave an optimal strategy for the case where the distance to the target is unknown. This result was rediscovered by Baeza-Yates et al. [4]. Additionally, the randomized strategy for linear search generalizes and gives an optimal strategy for this version too [17, 24, 25].

Baeza-Yates et al. [4] also considered the problem of finding a target with *integer coordinates* in the plane and presented various search strategies. When the distance to the target is known, it is also easy to get an optimal strategy. However, when the distance is unknown, and with no additional information available, no search strategy can have constant competitive ratio as there are $\Theta (n^2)$ integral points within distance at most *n* from the origin and any search strategy has to visit all of them in some order. For the natural extension of the problem in $\mathbb R^d$, the latter generalizes to any d≥3, as there are $\Theta (n^{d})$ integral points within distance at most *n* from the origin. When we are in $\mathbb R^d$ and the distance to the target is known, we hit a classical problem in Number Theory: on how many ways can we express a positive integer as sum of *d* squares of integers. For d≥5, there is no search strategy with constant competitive ratio as there are there are $\Omega (n^{d/2-1})$ integral points at distance exactly *n* from the origin; see, for example, Vaughan and Wooley [35].

In another variant of the problem in the plane by Gal [17], the searcher travels along a path until the target lies on the *segment* connecting the searcher’s current and starting positions, essentially sweeping around its starting position with an infinitely elastic cord until the target is swept. For this problem, Gal [17] gave a spiral search strategy achieving a competitive ratio of $17.289{\ldots }$, while Langetepe [29] showed that this ratio is optimal.

Hipke et al. [22] considered linear search when the target is at *distance* at least 1 and at most D≥1 from the starting point, where *D* is known at the start of the search. Bose et al. [10] provided a more careful analysis using the roots of a recursive sequence of polynomials and gave better lower and upper bounds on the competitive ratio with dependence on *D*. López-Ortiz and Schuierer [30] considered also this setting, for the case of concurrent rays. Compared to these works, there are two main differences in our work. Firstly and most importantly, we have a prediction all the way through the search, while they have a prediction only at the start. Secondly, we consider the problem in more general settings, namely in $\mathbb R^d$ for arbitrary *d*. An upper bound at the start does not suffice to find a point when when d>2 or when d=2 and c>1. (If d=2 and the exact distance is known, the problem can be easily solved since the target has to lie on a known circle, and that is the only additional instance that is solvable.)

Banerjee et al. [5] considered the problem of finding a target in a *graph* with information about the distance to the target. In their model, the target is at one vertex of the graph and each vertex has a value stored that is made available only when the searcher is at an adjacent vertex. For most vertices, the value stored at a vertex is the true distance from the vertex to the target, but for some vertices the value is wrong. Contrary to our setting, they do not assume a bounded error for the information at each vertex, but that the information is wrong at a bounded number of nodes. The bound on the number of nodes with wrong information then appears in the bound for the length of the search path.

Finally, Angelopoulos [2, 3] gave strategies for linear and multiple-ray search under a different model where a one-off, possibly erroneous hint or prediction on the target’s position is given at the start of the search. The prediction can be positional, directional, or, in general, a *k*-bit string encoding answers to *k* binary queries and the measure of the performance of a strategy is a trade-off between the competitive ratio under error-free prediction and that under erroneous prediction.

## Notation and Preliminaries

Since the dimension *d* is always fixed and clear from the context, we drop in the notation the dependency on *d*.

The ***ball*** centered at $p\in \mathbb R^d$ with radius *r* is $B(p,r)=\{ q\in \mathbb R^d\mid |pq|\le r \}$. We will also consider the ***spherical shells*** $S(p,r_1,r_2)= \{ q\in \mathbb R^d\mid r_1\le |pq|\le r_2 \}$. A spherical shell in the plane is an annulus. For a *c*-prediction function λ, whenever we are at a point $p\in \mathbb R^d$, we get a prediction λ(p) and we deduce that the target point $\boldsymbol{t}$ lies in the spherical shell $S(p,\lambda (p)/c, \lambda (p))$. See Fig. 4.

Note that we have made a modeling decision, namely we have assumed that there is an unknown function λ such that at each point *p* we get the prediction λ(p). In a more general setting, the *c*-prediction may not be a function, i.e., it could happen that we visit the same point *p* multiple times and at each time we get a different estimate of the distance from *p* to the target. However, getting a different estimate can only help, as it provides more information: if we get two different *c*-predictions λ and $\lambda^{\prime }$ at different times at the same point *p*, then we know that the target lies in the spherical shell $$S(p,\max \{\lambda ,\lambda '\}/c, \min \{\lambda ,\lambda '\})$$. This is more information than what we get if $$\lambda '=\lambda $$ because then we can only conclude that the target lies in $$S(p,\lambda /c,\lambda )$$, which is strictly larger. Thus, when searching for an optimal search strategy, we can assume that each time we visit the same point we get the same prediction. In particular, a search strategy could simply ignore the information obtained in the second and subsequent visits to the same point.

A ***path*** in $\mathbb R^d$ is a continuous function $\pi :[0,1] \rightarrow \mathbb R^d$. The paths in our search strategies will consist of an infinite number of straight-line segments and will exhibit a Zeno-like phenomena: they make an infinite number of turns in finite time and length. To show that the paths we define reach the target, we will use the following property, whose proof is an standard argument in continuity.

### Lemma 1

Let $\pi :[0,1] \rightarrow \mathbb R^d$ be a path. Assume that there is a point $p\in \mathbb R^d$ with the following property: for each ε>0 there exists some $\delta \in (0,1]$ such that the subpath $\pi ([1-\delta ,1])$ is contained in B(p,ε). Then π(1)=p, that is, *p* is the endpoint of the path π.

We will use ε-nets from metric space theory: An ε***-net*** for the ball *B*(*p*, *r*) is a subset *N* of points from *B*(*p*, *r*) such that (i) each point of *B*(*p*, *r*) is at distance at most ε from some point of *N*, and (ii) each two distinct points of *N* are at distance at least ε. Condition (i) can be equivalently be stated as $B(p,r)\subseteq \bigcup _{q\in N}B(q,\varepsilon )$. Condition (ii) is equivalent to telling that the balls B(q,ε/2), where q∈N, are pairwise interior disjoint.

The following bound on ε-nets follows from a well-known technique using volumes. We will not use the lower bound, but we include it to show that the upper bound is a reasonable estimate.

### Lemma 2

In $\mathbb R^d$, for each $\varepsilon \le r/2$, a ball of radius *r* has a ε-net with at least $(r/\varepsilon )^d$ and at most $(5r/2\varepsilon )^d$ elements.

### Proof

Using scaling and translation, it suffices to show the result for the unit ball centered at the origin, $B(\boldsymbol{o},1)$, and for $\varepsilon \le 1/2$.

Let *N* be an inclusion-wise maximal subset of points from $B(\boldsymbol{o},1)$ such that any two points are at distance at least ε. We claim that *N* is a ε-net. By definition, *N* satisfies property (ii) of the ε-net definition above. Since by maximality we cannot add any other point of $B(\boldsymbol{o},1)$ to *N*, each point of $B(\boldsymbol{o},1)$ has some point of *N* at distance at most ε, and thus property (i) is also satisfied.

It remains to bound from below and above the cardinality of *N*. Because of property (i), the ball $B(\boldsymbol{o},1)$ is contained in $\bigcup _{q\in N} B(q,\varepsilon )$. Using the constant $V_d=\tfrac{\pi ^{d/2}}{\Gamma (d/2+1)}$ that gives the volume of the unit ball in $\mathbb R^d$, we then have

$$ |N| \ge \frac{{{\,\textrm{vol}\,}}(B(\boldsymbol{o}, 1))}{{{\,\textrm{vol}\,}}(B(\boldsymbol{o},\varepsilon ))} = \frac{V_d}{V_d\cdot \varepsilon ^d} = \frac{1}{\varepsilon ^d}. $$

On the other hand, property (ii) implies that the family of balls $\{ B(q,\varepsilon /2) \mid q\in N\}$ are pairwise interior disjoint. Each such ball is contained in the enlarged ball $B(\boldsymbol{o}, 1+\varepsilon /2)$, and therefore

$$ |N| \le \frac{{{\,\textrm{vol}\,}}(B(\boldsymbol{o}, 1+\varepsilon /2))}{{{\,\textrm{vol}\,}}(B(\boldsymbol{o},\varepsilon /2))} = \frac{V_d \cdot \big ( (1+\varepsilon /2)^d\big )}{V_d\cdot (\varepsilon /2)^d} = \left( 1+\frac{2}{\varepsilon }\right) ^d \le \left( \frac{5}{2\varepsilon } \right) ^d, $$

where in the last inequality we used that $\varepsilon \le 1/2$. □

## Observations About the Problem Setup

First, it is important to note that a 2-dimensional disk cannot be covered by a curve of bounded length. This is definitely not surprising, but it requires a proof, as the so-called space filling curves [32] can cover a *d*-dimensional body for d≥2.

### Lemma 3

For each r>0 and each $p\in \mathbb R^2$, any curve that passes through each point of the 2-dimensional disk *B*(*p*, *r*) has infinite length.

### Proof

(Provided by Swanepoel [34].) Consider a rectangular, regular grid of n×n points inside *B*(*p*, *r*) with total side length Θ(r); the distance between any two neighbour grid points is Θ(r/n). Any curve that goes through those $n^2$ points has length Θ(rn) because the Euclidean minimum spanning tree for those points has length $(n^2-1)\cdot \Theta (r/n)= \Theta (rn)$. Sine *n* can be chosen arbitrarily large, for any number *L*, we can find a set of points that cannot be in the image of any path with length *L*. □

This result implies that, without additional information or constraints, a searcher cannot reach a target point $\mathbb R^d$ for d≥2, if it only knows that the target lies inside a ball of positive radius, no matter how small it is.

We say that a *c*-prediction function $\lambda \in \Lambda (\boldsymbol{t},c)$ is ***open*** if for all $p\ne \boldsymbol{t}$ we have $|p\boldsymbol{t}|< \lambda (p) < c\cdot |p\boldsymbol{t}|$. In other words, at each non-target point the prediction is not the correct distance and it is not the maximum possible it could be. This implies that $\boldsymbol{t}$ lies in the *interior* of the spherical shell $S(p,\lambda (p)/c, \lambda (p))$, which is an open set. For example, for c>1 the function $\lambda (p)=\tfrac{1+c}{2} \cdot |p\boldsymbol{t}|$ is open because $1< \tfrac{1+c}{2} < c$. Note that the definition is meaningful only for c>1. In the following we show that as long as the target has not been reached, there is a ball around it such that any point in the ball can still be the target: the predictions received at the points visited so far cannot distinguish among the candidate targets in that ball.

### Proposition 4

Assume that we have explored a compact set *X* of points of $$\mathbb R^d$$, that the target $$\boldsymbol{t}$$ does not belong to *X* and that the *c*-prediction λ is open and continuous. There exists an ε>0 such that the ball $$B(\boldsymbol{t},\varepsilon )$$ has the following property: for each $$\boldsymbol{t}'\in B(\boldsymbol{t},\varepsilon )$$, there is a *c*-prediction $\lambda^{\prime }$ in $$\Lambda (\boldsymbol{t}',c)$$ that is continuous, open and such that $$\lambda '(p)=\lambda (p)$$ for all p∈X.

Let us explain the necessity to assume that the prediction is *open*. This is not because the problem becomes easy otherwise. In general, without this restriction, predictions can still be such that there is an “uncertainty ball” which the target can lie anywhere in. However, it could also happen that for a *finite* set *X* of points we have that $\cap _{p\in X}S(p,\lambda (p)/c,\lambda (p))$ is a single point, which must then be the target $\boldsymbol{t}$. (This cannot happen when all predictions are open.) In such a case, if the searcher passes through all points of *X*, they have not reached the target but already have deduced its position. We further assume that *X* is compact and λ is continuous to avoid that such a deduction can be made when *X* is infinite.

### Proof of Proposition 4

**Fig. 2:**
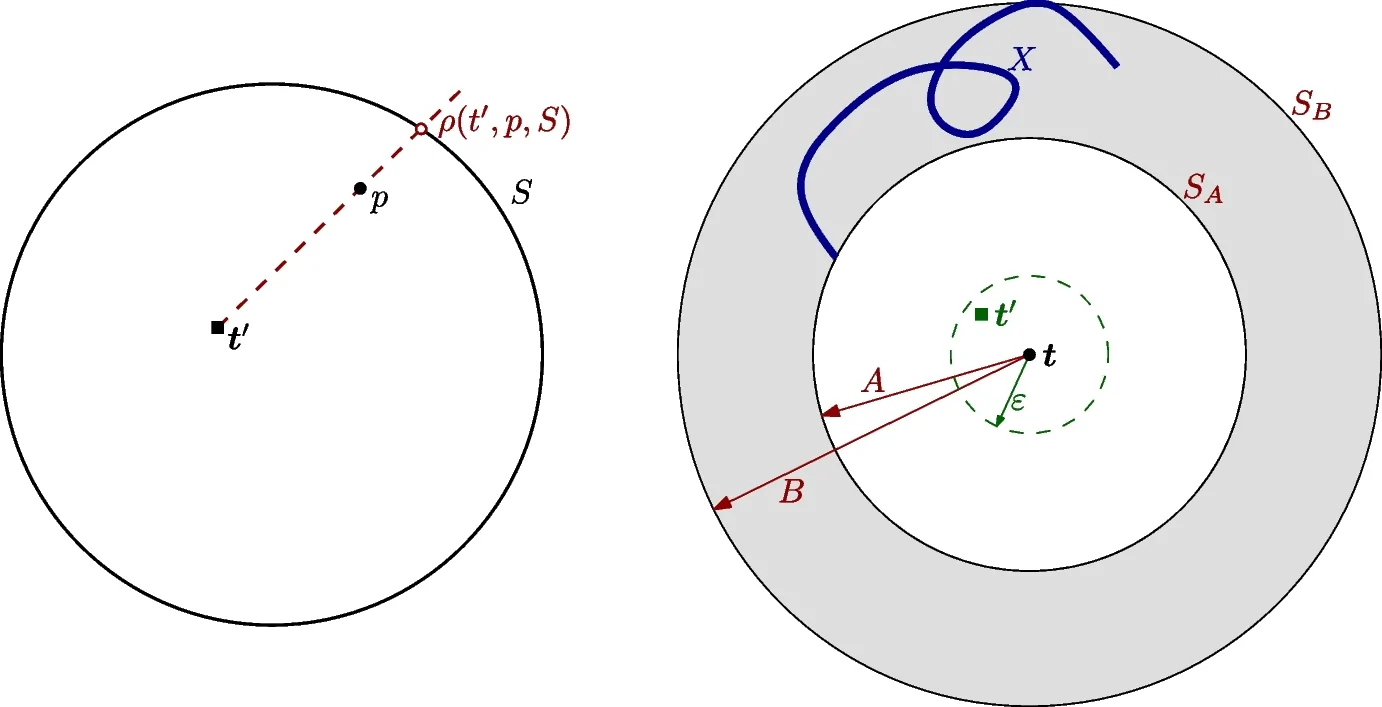
Proof of Proposition 4. Left: definition of $\rho (\boldsymbol{t}',p,S)$ to extend λ defined on *S*. Right: Main part of the proof

We first provide a simple extension tool. Let *S* be a sphere and let $$\boldsymbol{t}'$$ be a point in the interior of the ball bounded by *S*. Assume that we have a continuous and open *c*-prediction $$\lambda :S\rightarrow \mathbb R$$ for the potential target $$\boldsymbol{t}'$$, but defined only on *S*. Then there is a continuous and open *c*-prediction $\lambda^{\prime }$ for $$\boldsymbol{t}'$$ defined on the whole $$\mathbb R^d$$ that extends $\lambda^{\prime }$, that is, $$\lambda (p)=\lambda '(p)$$ for all p∈S. To show this, we introduce the following notation: for each point $$p\ne \boldsymbol{t}'$$, let $$\rho (\boldsymbol{t}',p,S)$$ be the unique point where the ray with origin at $$\boldsymbol{t}'$$ through *p* intersects the sphere *S*. See Fig. 2, left. We then define $$\lambda '(p):=\frac{|p\boldsymbol{t}'|}{|\rho (\boldsymbol{t}',p,S)\boldsymbol{t}'|} \cdot \lambda (\rho (t',p,S))$$ for all $$p\in \mathbb R^d\setminus \{ \boldsymbol{t}'\}$$, and $$\lambda '(\boldsymbol{t}'):=0$$. It is easy to see that $\lambda^{\prime }$ is an open *c*-prediction for $$\boldsymbol{t}'$$ because at each ray from $$\boldsymbol{t}'$$ it is just a linear function. It is also easy to see that $\lambda^{\prime }$ is continuous because λ is continuous. Finally, it is clear from the definition that $\lambda^{\prime }$ extends λ because for each p∈S we have $$p=\rho (\boldsymbol{t}',p,S)$$.

We now turn our attention to the main statement. Fix a *c*-prediction λ for the target $\boldsymbol{t}$ that is open and continuous. Figure 2, right, may help to follow the notation. Since *X* is compact and the distance function is continuous, we can define the values

$$ A:= \min \{ |p\boldsymbol{t}|\mid p\in X \} ~~\text { and }~~ B:= \max \{ |p\boldsymbol{t}|\mid p\in X \}. $$

They are both positive because $\boldsymbol{t}\notin X$, and we have $X\subseteq S(\boldsymbol{t},A,B)$. Because $\boldsymbol{t}\notin S(\boldsymbol{t},A,B)$ and $S(\boldsymbol{t},A,B)$ is compact, we can also define the values

$$ \varepsilon ':=\min \{ \lambda (p)-|p\boldsymbol{t}| \mid p\in S(\boldsymbol{t},A,B) \} ~~\text { and }~~ \varepsilon '':= \min \{ |p\boldsymbol{t}| - \lambda (p)/c \mid p\in S(\boldsymbol{t},A,B) \}. $$

Because λ is open, both values $\varepsilon^{\prime }$ and $\varepsilon^{\prime \prime }$ are strictly positive. Let $$\varepsilon =\min \{ \varepsilon ',\varepsilon ''\}/2$$, which is also strictly positive. We thus have

$$ \forall p\in S(\boldsymbol{t},A,B):~~~ |p\boldsymbol{t}|+\varepsilon< \lambda (p) < c \cdot (|p\boldsymbol{t}| - \varepsilon ). $$

We will show that all the points in the ball $B(\boldsymbol{t},\varepsilon )$ are possible targets. This is natural because each of those points is consistent with the information we got: the disk $B(\boldsymbol{t},\varepsilon )$ is contained in the spherical shell $S(p,\lambda (p)/c,\lambda (p))$ for all p∈X (and actually all $p\in S(\boldsymbol{t},A,B)$).

Fix any point $\boldsymbol{t}'\in B(\boldsymbol{t},\varepsilon )$. Note that for each $p\in S(\boldsymbol{t},A,B)$ we have

$$ \lambda (p) ~<~ c \cdot (|p\boldsymbol{t}| - \varepsilon ) ~\le ~ c \cdot (|p\boldsymbol{t}'|+|\boldsymbol{t}' \boldsymbol{t}| - \varepsilon ) ~\le ~ c \cdot (|p\boldsymbol{t}'|) $$

and

$$ \lambda (p) ~>~ |p\boldsymbol{t}| + \varepsilon ~\ge ~ |p\boldsymbol{t}'|-|\boldsymbol{t}'\boldsymbol{t}| +\varepsilon ~\ge ~ |p\boldsymbol{t}'|. $$

Therefore the restriction of λ to the spherical shell $S(\boldsymbol{t},A,B)$ is also an open and continuous *c*-prediction for $\boldsymbol{t}'$.

Let $S_A$ and $S_B$ be the inner and outer spheres bounding the spherical shell $S(\boldsymbol{t},A,B)$, respectively. Let $\lambda _A$ and $\lambda _B$ be the restriction of λ to $S_A$ and $S_B$, respectively. They are open and continuous because they are a restriction of λ.

As discussed at the beginning of the proof, we can extend $\lambda _A$ to an open and continuous *c*-prediction $\lambda '_A$ for the target $\boldsymbol{t}'$. Similarly, we can extend $\lambda _B$ to an open and continuous *c*-prediction $\lambda '_B$ for the target $\boldsymbol{t}'$.

Finally, we can define the function $\lambda '':\mathbb R^d\rightarrow \mathbb R$ by

$$ \lambda ''(p) ~=~ {\left\{ \begin{array}{ll} \lambda '_A(p), & \text {if } p\in B(\boldsymbol{t},A) \text {, ~~~[inside]}\\ \lambda (p), & \text {if } p\in S(\boldsymbol{t},A,B) \text {, ~~~[spherical shell]}\\ \lambda '_B(p), & \text {if } p\notin B(\boldsymbol{t},B) \text { ~~~[outside].}\\ \end{array}\right. } $$

This function $\lambda^{\prime \prime }$ is continuous because $$\lambda '_A(p)=\lambda (p)$$ for all $$p\in S_A$$, $$\lambda '_B(p)=\lambda (p)$$ for all $$p\in S_B$$, and because $$\lambda ,\lambda '_A,\lambda '_B$$ are continuous. It is also open and a *c*-prediction because each of the functions $$\lambda ,\lambda '_A,\lambda '_B$$ has these properties. Finally, it is obvious that $$\lambda (p)=\lambda ''(p)$$ for all p∈X because $$X\subseteq S(\boldsymbol{t},A,B)$$. □

Proposition 4 implies that any search strategy that reaches the target for all *c*-predictions, where c>1, cannot consist of a finite number of pieces. Indeed, for any *c*-prediction λ that is open and continuous, until we do not reach the target, there is a ball $$B_\varepsilon $$ of radius ε>0 such that the target may be any point of $$B_\varepsilon $$, and the information collected so far cannot distinguish among the possible targets. In particular, an adversary could change from one continuous and open $$\lambda \in \Lambda (\boldsymbol{t},c)$$ to another continuous and open $$\lambda '\in \Lambda (\boldsymbol{t}',c)$$, for a suitable $$\boldsymbol{t}'\ne \boldsymbol{t}$$, at any time before reaching the target because λ and $\lambda^{\prime }$ agree on the points that have been explored so far and are indistinguishable. Moreover, any search path needs to have an infinite number of pieces because an adversary can change the target at any time during the search. Finally, knowing that the prediction λ is continuous does not help.

Note also that, because of Lemma 3, we cannot cover all those candidate targets of $B_\varepsilon$ with a curve of bounded length, unless we collect additional information. This means that we cannot have a search strategy that ignores the prediction λ after some time, because from that moment all points in a ball of positive radius keep being possible targets, and we cannot visit all of them with a curve of bounded length that neglects additional information.

Let us consider for a moment a variant of the model, discussed in Sect. 1.2, where the searcher travels at unit speed and gets the value λ(p) for the current position *p* at regular time intervals. Since we have argued that the searcher needs to get λ(p) at an infinite number of points *p*, it follows that in this variant the target cannot be reached within any bounded time. (However, if the target is known to have integer coordinates, this argument does not hold anymore and how to achieve bounded competitive ratio is discussed in Sect. 4.1.)

We return now to our main, continuous model. The following property shows that from the data collected at any given point, we can infer another *c*-prediction function using the triangular inequality. Moreover, the part that we infer is 1-Lipschitz and thus continuous. The following result is not used in our work, but it may be useful to future research.

### Lemma 5

Let $X\subset \mathbb R^d$ be any non-empty set of points and λ a *c*-prediction for target $\boldsymbol{t}$. Then, the function $\tilde{\lambda }$ defined by

$$ p\in \mathbb R^d ~\mapsto ~ \tilde{\lambda }(p)= \inf \{ |pp'|+\lambda (p') \mid p'\in X \} $$

is 1-Lipschitz and the function

$$ p\in \mathbb R^d ~\mapsto ~ \min \{ \lambda (p), \tilde{\lambda }(p)\} $$

is a *c*-prediction for $\boldsymbol{t}$.

### Proof

The idea is that for any $p^{\prime }\in X$, we can replace the prediction at *p* by $$\min \{|pp'|+\lambda (p'), \lambda (p) \}$$, which is something we can deduce because of the triangular inequality.

We first show that the function $\tilde{\lambda }$ is 1-Lipschitz. Note that $\min \{ |pp'|+\lambda (p') \mid p'\in X \}$ is not well-defined; the minimum may not exist because we do not assume anything about the prediction function λ or the set *X*. This is the reason that in the definition of $\tilde{\lambda }$ we are using the infimum. For each ε>0 and each $p\in \mathbb R^d$, there exists a point $p'_\varepsilon \in X$ such that

$$ |pp'_\varepsilon |+\lambda (p'_\varepsilon ) ~\le ~ \tilde{\lambda }(p) + \varepsilon . $$

Then

$$\begin{aligned} \forall p,q\in \mathbb R^d:~~~~ \tilde{\lambda }(q) - \tilde{\lambda }(p)&\le \big ( |qp'_\varepsilon | + \lambda (p'_\varepsilon )\big ) - \big ( |pp'_\varepsilon | + \lambda (p'_\varepsilon ) -\varepsilon \big )\\&= |qp'_\varepsilon | - |pp'_\varepsilon | +\varepsilon \\&\le |qp|+\varepsilon . \end{aligned}$$

Because of symmetry we obtain that

$$ \forall \varepsilon >0, \forall p,q\in \mathbb R^d:~~~~ |\tilde{\lambda }(p) - \tilde{\lambda }(q)| \le |pq|+\varepsilon . $$

This implies that

$$\begin{aligned} \forall p,q\in \mathbb R^d&:~~~~ |\tilde{\lambda }(p) - \tilde{\lambda }(q)| \le |pq| \end{aligned}$$

and therefore $\tilde{\lambda }$ is 1-Lipschitz. Note that, in general, $\tilde{\lambda }$ is *not* a *c*-prediction function because, if the target does not belong to *X*, it is always non-zero.

Next we show that the function $\mu (p):=\min \{ \lambda (p), \tilde{\lambda }(p)\}$ is a *c*-prediction for $\boldsymbol{t}$. For this we use that λ is a *c*-prediction for $\boldsymbol{t}$. On the one hand we have

$$ \forall p\in \mathbb R^d:~~~ \mu (p) ~=~ \min \{ \lambda (p), \tilde{\lambda }(p) \} ~\le ~ \lambda (p) ~\le ~ c\cdot |p\boldsymbol{t}|. $$

On the other hand

$$\begin{aligned} \forall p\in \mathbb R^d:~~~ \tilde{\lambda }(p) ~&=~ \inf \{ |pp'|+\lambda (p') \mid p'\in X \} ~\ge ~ \inf \{ |pp'|+|p'\boldsymbol{t}| \mid p'\in X \} \\ ~&\ge ~ \inf \{ |p\boldsymbol{t}| \mid p'\in X \} ~\ge ~ |p\boldsymbol{t}|, \end{aligned}$$

and therefore

$$\begin{aligned} \forall p\in \mathbb R^d:~~~ \mu (p) ~=~ \min \{ \lambda (p), \tilde{\lambda }(p) \} ~\ge ~ \min \{ |p\boldsymbol{t}|, |p\boldsymbol{t}| \} ~=~ |p\boldsymbol{t}|. \end{aligned}$$

This shows that $\mu (\cdot )$ is a *c*-prediction for $\boldsymbol{t}$, as claimed. □

The relevance of the result is that at any given point *p* we get two predictions, λ(p) and the value $\tilde{\lambda }(p)$ that we can infer. When they are distinct, we can infer more information, which can help the search. However, as we mentioned, we do not exploit this in the strategies we describe.

Finally, we note that the case of c=1 is easy.

### Proposition 6

(Case c=1) For 1-predictions in $\mathbb R^d$, there is a search strategy with competitive ratio 1+ε for each ε>0.

### Proof

As usual, we assume that the search starts at the origin $\boldsymbol{o}$. When c=1, whenever we query for the distance at a point *p*, we get $\lambda (p)=|p\boldsymbol{t}|$ and we conclude that the target lies on the d-1 sphere centered at *p* with radius λ(p). In particular, $\lambda (\boldsymbol{o})=|\boldsymbol{o}\boldsymbol{t}|$.

We have to find a strategy that reaches the target using a path of length at most $(1+\varepsilon )|\boldsymbol{o}\boldsymbol{t}|=(1+\varepsilon )\lambda (\boldsymbol{o})$. If $\lambda (\boldsymbol{o})=0$, we are already at the target. We thus assume that $\lambda (\boldsymbol{o})\ne 0$.

**Fig. 3:**
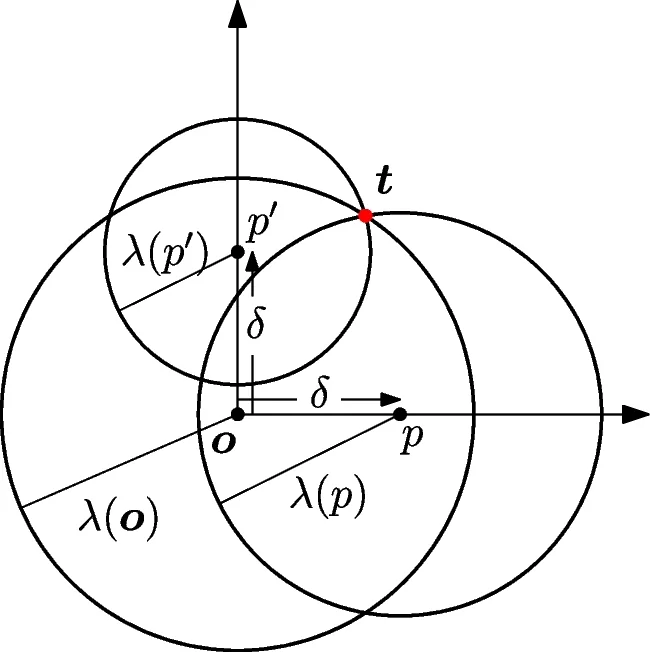
Predictions at the origin $$\boldsymbol{o}$$ and at two points *p*, and $p^{\prime }$, one on each axis and at distance δ from the origin. (Here δ is larger than in the proof of Proposition 6 for better visual clarity.) The target $$\boldsymbol{t}$$ must lie in the intersection of the three corresponding circles

For each of the main orthogonal axis, we move $\delta =\lambda (\boldsymbol{o})\cdot \varepsilon /(2d)$ along the axis, query the distance to the target, and go back to the origin. The target has to lie in the intersection of the *d* spheres centered at the points where we queried and also at the sphere centered at the origin. The intersection of these d+1 spheres is always a single point, which must be $\boldsymbol{t}$; see Fig. 3 for an example for d=2. The uniqueness of the point can be seen for example using the lifting to the paraboloid in $\mathbb R^{d+1}$ defined by the function $(x_1,\dots ,x_d)\in \mathbb R^d \mapsto \left( x_1,\dots ,x_d, \sum _{i=1}^d (x_i)^2\right) \in \mathbb R^{d+1}$. Each of those spheres corresponds to the intersection of a hyperplane with the paraboloid, and the normals of those d+1 hyperplanes are linearly independent. Therefore, the intersection of those hyperplanes is a single point, which by construction must lie on the paraboloid.

We have walked exactly $(2d)\delta =\varepsilon |\boldsymbol{o}\boldsymbol{t}|$, came back to the origin, and we know now the exact position of the target. Walking to the target takes additional $|\boldsymbol{o}\boldsymbol{t}|$ length.□

## Upper Bound

In this section, we provide search strategies to reach the target in $\mathbb R^d$ when we have a *c*-prediction. First, we provide the key lemma that tells us how to get a sequence of points whose λ-values decreases geometrically. Then, we provide a strategy when the prediction factor is known, followed by a strategy to handle the case for unknown prediction factor. In this setting, we adapt the notation so that $c^*$ is the true prediction factor, while *c* is a guess for the true prediction factor. At the end of the section, we discuss some extensions.

### Lemma 7

Assume that we are at point $p_i$ and the prediction factor $c^*$ is perhaps unknown. Let c≥1 be a guess for $c^*$. Using a search through a path $\gamma _{i+1}$ of length at most $2(5c)^d\cdot \lambda (p_i)$ we get to one of the following outcomes:

- we move from $p_i$ to a point $p_{i+1}$ such that $\lambda (p_{i+1})\le \lambda (p_i)/2$, or
- we come back to $p_i$ and correctly deduce that $c<c^*$.

Moreover, all points of the path $\gamma _{i+1}$ are at distance at most $2\lambda (p_i)$ from $\boldsymbol{t}$.

The length of the path $\gamma _{i+1}$ can be improved to $O(c^{d-1})\cdot \lambda (p_i)$.

### Proof

**Fig. 4:**
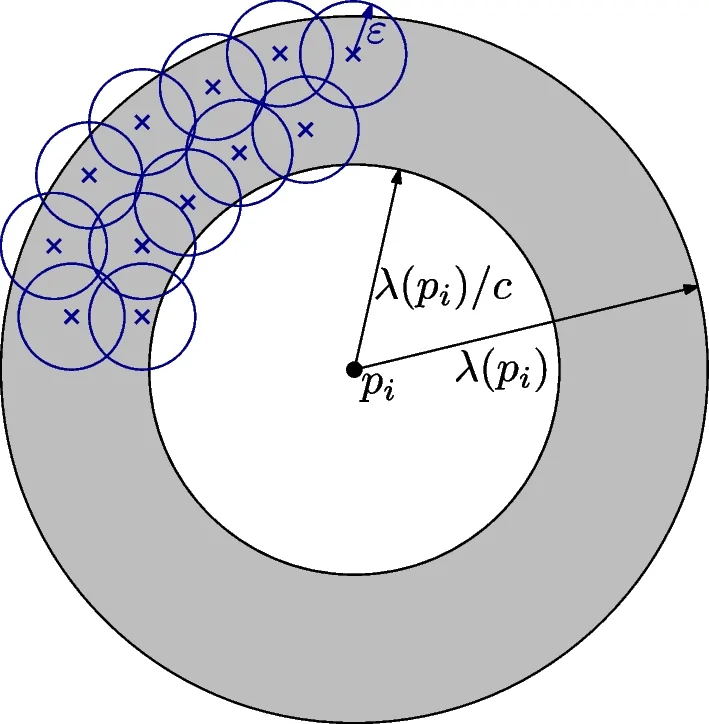
Spherical shell $S(p_i,\lambda (p_i)/c,\lambda (p_i))$ where $\boldsymbol{t}$ must lie and (part of) a net for the shell

Recall that $|p_i\boldsymbol{t}| \le \lambda (p_i)$. Set $\varepsilon =\tfrac{\lambda (p_i)}{2c} \le \tfrac{\lambda (p_i)}{2}$ and let *N* be a ε-net for the ball $B(p_i,\lambda (p_i))$ given by Lemma 2. See Fig. 4. Note that

$$ |N| \le \left( \frac{5\lambda (p_i)}{2\varepsilon }\right) ^d = (5c)^d. $$

Since the target is contained in $B(p_i,\lambda (p_i))$ and *N* is a ε-net, there is some point $p_*\in N$ such that $|p_*\boldsymbol{t}|\le \varepsilon = \lambda (p_i)/2c$. If $c^*\le c$, then we have $\lambda (p_*) \le c^*\cdot |p_*\boldsymbol{t}| \le c^* \cdot \lambda (p_i)/2c \le \lambda (p_i)/2$. If $c^* > c$, then we have no guaranteed useful bound for $\lambda (p_*)$.

Our search path will go through the points of *N* in some order and, at each point of *N*, we query for the prediction $\lambda (\cdot )$.

We finish the path as soon as we reach some point $q_*\in N$ such that $\lambda (q_*)\le \lambda (p_i)/2$. If for all the points *p* of *N* we have $\lambda (p)> \lambda (p_i)/2$, then we go back to the point $p_i$. This finishes the general description of the path $\gamma _{i+1}$.

In the first case, we set $p_{i+1}=q_*$ and we have moved to a point $p_{i+1}$ with $\lambda (p_{i+1})\le \lambda (p_i)/2$. When $c^*\le c$, we have to be in this case since the point $p_*$ satisfies the stopping condition $\lambda (p_*)\le \lambda (p_i)/2$. Thus, if we do not have such a point, we can conclude that $c^*>c$.

Let’s assume that $\gamma _{i+1}$ goes through the points of *N* in arbitrary order. This gives us a simple bound on $\gamma _{i+1}$’s length, which has concrete constants and is also easier to use in computations in subsequent theorems.

First, note that any two points in $B(p_i,\lambda (p_i))$ are at distance at most $2\lambda (p_i)$. Furthermore, the first edge has length at most $\lambda (p_i)$ and, if $\gamma _{i+1}$ comes back to $p_i$, also the last edge has length at most $\lambda (p_i)$. Using the bound of Lemma 2 for |*N*|, we get that the path $\gamma _{i+1}$ has length at most

$$ \lambda (p_i) + (|N|-1)\cdot (2\lambda (p_i)) + \lambda (p_i) \le 2\cdot |N|\cdot \lambda (p_i) \le 2(5c)^d \cdot \lambda (p_i). $$

Alternatively, it is known [14] that for any set of *n* points in the *d*-dimensional ball *B*(*p*, *r*) there is a tour visiting them with length

$$ r\cdot \left( d \big (2(d-1) \big )^{\frac{1-d}{2d}} \cdot n^{1-\frac{1}{d}} + O\big ( n^{1-\frac{2}{d}} \big ) \right) =r\cdot O\big (n^{\frac{d-1}{d}}\big ). $$

(Unfortunately, the constant hidden in the term $O\big ( n^{1-\frac{2}{d}} \big )$ is not given explicitly in [14].) This implies that we can also use a path $\gamma _{i+1}$ of length $\lambda (p_i) \cdot O\big ( |N|^{\frac{d-1}{d}}\big ) = O(c^{d-1})\cdot \lambda (p_i)$. With this, the dependency on *c* is slightly better at the expense of having more ugly-looking constants hidden in the *O*-notation.

Finally, we note that the whole path $\gamma _{i+1}$ is contained in $B(p_i,\lambda (p_i))$, which is contained in $B(\boldsymbol{t},2\lambda (p_i))$ because $\boldsymbol{t}\in B(p_i,\lambda (p_i))$. □

### Theorem 8

(Known prediction factor) Consider the search with predictions problem in $\mathbb R^d$ where the prediction factor $c^*>1$ is known. There is a search strategy to reach the target with competitive ratio $4\cdot 5^d\cdot (c^*)^{d+1}$. The competitive ratio of the strategy can be improved to $O((c^*)^d)$.

### Proof

Let $p_0=\boldsymbol{o}$ be the starting point and recall that $\lambda (p_0) \le c^* \cdot |\boldsymbol{o}\boldsymbol{t}|$.

For i=0,1,2⋯ iteratively, we use Lemma 7 with the guessed prediction factor $c=c^*$ to obtain a path $\gamma _{i+1}$. As the prediction factor is correct, we always have the outcome in the first item: $\gamma _{i+1}$ finishes at a point $p_{i+1}$ with $\lambda (p_{i+1})\le \lambda (p_i)/2$. It follows by induction that for each $i\in \mathbb N\cup \{ 0 \}$ we have $\lambda (p_i)\le \lambda (p_0)/2^i$ and therefore

$$ {{\,\textrm{len}\,}}(\gamma _{i+1}) ~\le ~ 2(5c^*)^d\cdot \lambda (p_i) ~\le ~ 2(5c^*)^d\cdot \frac{\lambda (p_0)}{2^i}. $$

Let γ be the concatenation of the paths $\gamma _1,\gamma _2,\dots$. Then

$$\begin{aligned} {{\,\textrm{len}\,}}(\gamma ) ~&=~ \sum _{i=0}^\infty {{\,\textrm{len}\,}}(\gamma _{i+1}) ~\le ~ \sum _{i=0}^\infty \left( 2(5c^*)^d\cdot \frac{\lambda (p_0)}{2^i} \right) ~=~ 4(5c^*)^d \cdot \lambda (p_0) \\&\le ~ 4 \cdot 5^d (c^*)^{d+1} \cdot |\boldsymbol{o}\boldsymbol{t}|. \end{aligned}$$

If one uses the alternative bound on ${{\,\textrm{len}\,}}(\gamma _{i+1})$ in Lemma 7, i.e., ${{\,\textrm{len}\,}}(\gamma _{i+1})= O((c^*)^{d-1})\cdot \lambda (p_i)$, a similar calculation to the above gives that ${{\,\textrm{len}\,}}(\gamma ) = O((c^*)^d) \cdot |\boldsymbol{o}\boldsymbol{t}|$.

The path makes an infinite number of straight-line moves. Since for each $i\in \mathbb N$, the suffix of the path γ after $p_{i+1}$ is at distance at most $2\cdot \lambda (p_{i+1}) \le 2 \cdot \lambda (p_0)/2^{i+1}= \lambda (p_0)/2^i$ from $\boldsymbol{t}$, Lemma 1 implies that $\boldsymbol{t}$ is the endpoint of the path γ. □

### Theorem 9

(Unknown prediction factor) Consider the search with predictions problem in $\mathbb R^d$ where the prediction factor $c^*$ is unknown. There is a search strategy to reach the target with competitive ratio $6\cdot 10^d \cdot (c^*)^{d+1}$. The competitive ratio of the strategy can be improved to $O((c^*)^d)$.

### Proof

The basic idea is using an exponential search for the constant *c*. At each step we use Lemma 7 to either move to a point with smaller predicted distance to the target or to detect that our guess for *c* is too small and double it. Index *j* parameterizes the current guess $c=2^j$, and $p^{(j)}_i$ denotes a point during that guess. At each step we will increase either *i* or *j*.

We start setting j=1, i=0 and $p^{(j)}_i=p^{(1)}_0=\boldsymbol{o}$.

**Fig. 5:**
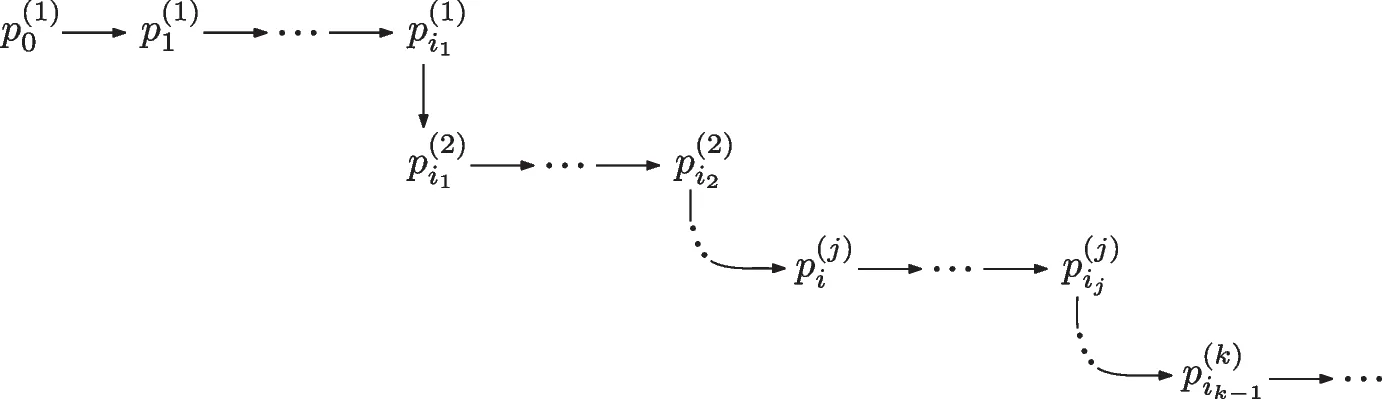
Visualizing the sequence of points $\Big ( \bigl ( p^{(j)}_i \bigr )_{i\in I_j}\Bigr )_{j\in J}$. When increasing *i* we move right, when increasing *j* we move down. Here, we are using k=max(J) and $i_j=\max (I_j)$ for all j∈J

From the current point $p^{(j)}_i$, we use Lemma 7 with the guessed prediction factor $c=2^j$ to obtain a path $\gamma ^{(j)}_{i+1}$. If the outcome is given by the first item of Lemma 7, then we get to a point $p^{(j)}_{i+1}$ with $\lambda (p^{(j)}_{i+1})\le \lambda (p^{(j)}_i)/2$; in this case we increase *i*. If the outcome is given by the second item of Lemma 7, then we get back to $p^{(j)}_i$; in this case we set $p^{(j+1)}_i=p^{(j)}_i$, increase *j* and, from this point on, we will use the new guessed prediction factor $2^j$, which is twice larger than before. See Fig. 5 for an schematic view of the sequence of points.

Let *k* be the largest value that index *j* takes through the procedure. Note that $k\le \lceil \log _2 c^* \rceil$ since $2^{\lceil \log _2 c^* \rceil }$ is an overestimate to $c^*$. If we arrive to $k= \lceil \log _2 c^* \rceil$, from that point on we will always extend the search path using the outcome in the first item of Lemma 7. Set $J=\{ 1,\dots , k\}$, which is the set of values that index *j* takes through the procedure.

For each j∈J, let $I_j$ be the set of indices *i* such that the point $p^{(j)}_i$ is defined and let $i_j=\max (I_j)$. Note that $i_k$ is undefined because $I_k$ is infinite, but $i_j$ is defined for all j<k because $I_j$ is finite for all j<k. By construction, the index $i_j$ is the first element of $I_{j+1}$, for all j<k. It may happen that, for some j<k, the set $I_j$ contains a single element. This happens when *j* is increased in successive steps and thus $p^{(j)}_i=p^{(j+1)}_i=p^{(j+2)}_i$.

It follows by induction that, for all j∈J and all $i\in I_j$, we have $\lambda (p^{(j)}_i)\le \lambda (p^{(1)}_0)/2^i = \lambda (\boldsymbol{o})/2^i$. Note that this bound is independent of the index *j*. Therefore

$$ \forall j\in J, ~ i\in I_j:~~~ {{\,\textrm{len}\,}}(\gamma ^{(j)}_{i+1})\le 2(5\cdot 2^j)^d\cdot \lambda (p^{(j)}_i) \le 2(5\cdot 2^j)^d \cdot \frac{\lambda (\boldsymbol{o})}{2^i}. $$

Let γ be the concatenation of the paths $\gamma ^{(j)}_{i+1}$, in the same order as we constructed them: first $\gamma ^{(1)}_{i+1}$ for increasing $i\in I_1$, then $\gamma ^{(2)}_{i+1}$ for increasing $i\in I_2$, and so on until we reach the infinite sequence $\gamma ^{(k)}_{i+1}$ for increasing $i\in I_k$. We then have

$$\begin{aligned} {{\,\textrm{len}\,}}(\gamma ) ~&=~ \sum _{j=1}^{k} \sum _{i\in I_j}{{\,\textrm{len}\,}}(\gamma ^{(j)}_{i+1}) ~\le ~ \sum _{j=1}^{k} \sum _{i\in I_j}\left( 2(5\cdot 2^j)^d\cdot \frac{\lambda (\boldsymbol{o})}{2^i}\right) . \end{aligned}$$

Using that for all j<k the sets $I_j$ and $I_{j+1}$ have only $i_j=\max (I_j)$ in common, that $2^j\le c^*$ for all j<k, that $2^{k}< 2c^*$, and that $\lambda (\boldsymbol{o}) \le c^* \cdot |\boldsymbol{o}\boldsymbol{t}|$, we get

$$\begin{aligned} {{\,\textrm{len}\,}}(\gamma ) ~&\le ~ \sum _{j=1}^{k-1} \left( 2(5\cdot 2^j)^d\cdot \frac{\lambda (\boldsymbol{o})}{2^{i_j}} \right) + \sum _{i=0}^\infty \left( 2(5\cdot 2c^*)^d\cdot \frac{\lambda (\boldsymbol{o})}{2^i}\right) \\  &\le ~ 2\cdot 5^d\cdot \lambda (\boldsymbol{o}) \cdot \sum _{j=1}^{k-1} (2^d)^j + 4(10 c^*)^d\cdot \lambda (\boldsymbol{o}) \\&=~ 2\cdot 5^d\cdot \lambda (\boldsymbol{o}) \cdot \frac{(2^d)^k- 2^d}{2^d-1} + 4(10 c^*)^d\cdot \lambda (\boldsymbol{o}) \\&\le ~ 2\cdot 5^d\cdot \lambda (\boldsymbol{o}) \cdot \frac{(2c^*)^d - 2^d}{2^d-1} + 4(10 c^*)^d\cdot \lambda (\boldsymbol{o}) \\&\le ~ 2\cdot 5^d\cdot \lambda (\boldsymbol{o}) \cdot 2(c^*)^d + 4(10 c^*)^d\cdot \lambda (\boldsymbol{o}) \\  &=~ 4\cdot 5^d\cdot (c^*)^d \cdot \lambda (\boldsymbol{o}) \cdot (1+2^d) \\  &\le ~ 4\cdot 5^d\cdot (c^*)^d \cdot \lambda (\boldsymbol{o}) \cdot (\tfrac{3}{2}\cdot 2^d ) \\  &\le ~ 6\cdot 10^d \cdot (c^*)^{d+1} \cdot |\boldsymbol{o}\boldsymbol{t}|. \end{aligned}$$

If one uses the alternative bound on ${{\,\textrm{len}\,}}(\gamma ^{(j)}_{i+1})$ from Lemma 7 (with $c=2^j$), i.e., ${{\,\textrm{len}\,}}(\gamma ^{(j)}_{i+1}) = O((5 \cdot 2^j)^{d-1})\cdot \lambda (p^{(j)}_i)$, a similar calculation to the above gives that ${{\,\textrm{len}\,}}(\gamma ) = O((c^*)^d) \cdot |\boldsymbol{o}\boldsymbol{t}|$.

The path makes an infinite number of straight-line moves. Since for each $i\in I_k$, the suffix of the path γ after $p^{(k)}_{i+1}$ is at distance at most $2\cdot \lambda (p^{(k)}_{i+1}) \le 2 \cdot \lambda (\boldsymbol{o})/2^{i+1}= \lambda (\boldsymbol{o})/2^i$ from $\boldsymbol{t}$, Lemma 1 implies that $\boldsymbol{t}$ is the endpoint of the path γ. □

### Extensions

First, the same approach works for spaces of bounded doubling dimension [19, 21], as long as there is a concept of path to connect points such that the length of the path is the same as the distance between the points. We could also change the setting so that the cost of moving from one point to another is the distance between the points. For this, one has to use ε-nets in spaces of bounded doubling dimension [20].

When the target is known to have integral coordinates, we can modify the search so that it has a finite number of segments. Indeed, as soon as we reach a point $p_i$ such that $\lambda (p_i)< 1/2$, we know that the target is at distance smaller than 1/2 from $p_i$ and there is a unique point with integral coordinates in $B(p_i,\lambda (p_i))$. We can then just move to that point. A similar approach works when the target is known to have coordinates with bounded resolution by scaling the setting. In the case where we can detect the target when we are within a given distance δ, we can finish the search when we reach a point $p_i$ with $\lambda (p_i) \le \delta$, thus, also bounding the number of steps (which will depend on the initial estimate $\lambda (p_0)$). In all these special cases, for both known and unknown prediction factor, the search path γ is shorter than the one in the general case by at most a constant factor (i.e., the tail in the infinite sums in the proofs of  Theorems 8 and 9), which depends only on $c^*$ and *d*. Therefore, the competitive ratios of the strategies stay the same. As noted in Sect. 1.3, without predictions or any additional information about the target, even for searching on the integer grid, there is no strategy with constant competitive ratio for any d≥2 (respectively d≥5) when the distance to target is unknown (known).

Let us consider now the variant of the model where the searcher travels at unit speed and gets the value λ(p) for the current position *p* at regular time intervals. We have shown in Sect. 3 (see the discussion after Proposition 4) that the searcher cannot reach the target in bounded time. However, if the target is known to have integer coordinates or the target is detected when we are within a given distance δ, then our search strategies can be adapted to reach the target. For concreteness, consider the case where the target is known to have integer coordinates and the searcher gets λ(p) for the current position after each time unit. This means that, before detecting the target, we have $1/2 \le \lambda (p)$ for all points *p* where the value λ(p) is obtained. The first bound for the length of the path obtained in Lemma 7 is at least $2(5c)^d \cdot (1/2) = (5c)^d$. We modify the search strategy in the proof of Lemma 7 in the naive way: when the searcher reaches a point *p* in the net *N*, it waits until the value λ(p) is revealed, which takes at most a unit of time. This increases the time spent by at most $|N|\le (5c)^d$. Therefore, in each use of Lemma 7 we use twice more time that the time bound that was guaranteed before, when the waiting at each each net point was not needed. It follows that the guaranteed bound on the time used by the searcher and the guaranteed competitive ratio is twice larger than before. A similar bound can be obtained when detection is guaranteed if the searcher is at distance at most δ from the target, but in this case the factor 1/δ shows up in the competitive ratio.

Our strategies work also in the case where predictions may additionally underestimate the actual distance to the target by, say, some factor $c^{\prime }\leq 1$, i.e., $$c' \cdot |p\boldsymbol{t}|\le \lambda (p)$$. Then, when both factors $c,c^{\prime }$ are known, this is equivalent to scaling up the prediction by $1/c^{\prime }$ to get a new one with factor $c/c^{\prime }$. When the factors are unknown, the strategy in Theorem 9 still visits points with geometrically decreasing predictions and since for each point, say $$p_i$$, we now have that $$|p_i\boldsymbol{t}| \le \lambda (p_0)/(2^i \cdot c')$$ the path converges to the target (as $c^{\prime }$ is constant).

One can consider the version when the target is an unknown *k*-flat *F* in $\mathbb R^d$. The same strategies work, also if we do not know the prediction factor nor the dimension, *k*, of the flat. Indeed, we are constructing a path as a concatenation of segments such that the distance to *F* gets arbitrarily small for each suffix of the path. Formally, for each ε>0, there is a suffix of the path such that all the points on the path are at distance ε from *F*. Since the path is continuous and has bounded length, the endpoint of the path has to be at distance 0 from *F*.

Finally, note that for small *d* there are tighter bounds on the size of ε-nets translating to better constants in our approach. For c≈1 one can also exploit that we need an ε-net of the spherical shell $S(\boldsymbol{o},r/c,r)$, which is much smaller than the whole ball $S(\boldsymbol{o},r)$. However, this improvement is expected to be small since even the ε-nets for spheres are not much better.

## Lower Bound

In this section, we provide a lower bound for the search problem with *c*-predictions. Our lower bounds are meaningful when we assume that *c* is large enough, i.e., c>2. The idea is to construct *c*-predictions for infinitely many different targets with the property that any two of them are indistinguishable, unless we are quite close to the target. For each such prediction λ, there is a small ball around the target where the value of λ is different from the other predictions, but most of the other predictions (except λ) have the same value on that small ball. Then, the searcher has to visit *all* the small balls around the targets, because in the worst case the target is going to be in the last small ball that is visited.

First, inspired by the technique by Elbassioni, Fishkin and Sitters [13] developed for the Euclidean Traveling Salesperson with Neighbourhoods, one can show the following bound. See Dumitrescu and Tóth [12] for an improvement over [13] where the same idea is reused.

### Lemma 10

For any given radius $\delta \le 1/2$ and dimension d≥1, consider the infinite family of balls

$$ \mathbb B\!=\! \big \{ B(p,\delta )\subset \mathbb R^d\mid p\in B(\boldsymbol{o},1/2-\delta )\big \} \!=\! \big \{ B(p,\delta )\subset \mathbb R^d\mid B(p,\delta ) \!\subseteq \! B(\boldsymbol{o},1/2)\big \}.$$

Each path in $\mathbb R^d$ that contains at least one point from each ball of B has length at least $\left( \big (\frac{1}{2\delta }-1 \big )^d - 1\right) \cdot \delta \cdot \sqrt{\frac{\pi }{d}}$.

### Proof

For each r≥0, let $V_d(r)$ denote the volume of the *d*-dimensional ball of radius *r*. It is clear that $V_d(r)=r^d\cdot V_d(1)$.

Consider any path π that touches each ball of B and let *L* be its length. Consider the volume of the Minkowski sum $\pi \oplus B(\boldsymbol{o},\delta )$ of the (points on the) path π and the ball $B(\boldsymbol{o},\delta )$, that is the set of points at distance at most δ from π. Since the path touches each ball of B, the set $\pi \oplus B(\boldsymbol{o},\delta )$ contains the center of each ball of B. This means that $\pi \oplus B(\boldsymbol{o},\delta )$ contains $B(\boldsymbol{o},1/2-\delta )$, and therefore we have

$$ {{\,\textrm{vol}\,}}\big ( \pi \oplus B(\boldsymbol{o},\delta ) \big ) ~\ge ~ {{\,\textrm{vol}\,}}\big ( B(\boldsymbol{o},1/2-\delta )\big ) ~=~ \left( \frac{1}{2}-\delta \right) ^d\cdot V_d(1). $$

On the other hand, for each path π of length *L*, we have

$$ {{\,\textrm{vol}\,}}\big ( \pi \oplus B(\boldsymbol{o},\delta ) \big ) ~\le ~ V_d(\delta ) + L \cdot V_{d-1}(\delta ) ~=~ \delta ^d \cdot V_d(1) + L \cdot \delta ^{d-1} \cdot V_{d-1}(1), $$

with equality if and only if π is a straight line. See for example Lemmas 5.1 and 5.2 in [12]. Therefore we get

$$ L ~\ge ~ \frac{\left( \frac{1}{2}-\delta \right) ^d\cdot V_d(1) - \delta ^d \cdot V_d(1)}{\delta ^{d-1} \cdot V_{d-1}(1)} ~=~ \left( \Big (\frac{1}{2\delta }-1 \Big )^d - 1\right) \cdot \delta \cdot \frac{V_d(1)}{V_{d-1}(1)}. $$

Using standard formulas for the volume of the *d*-dimensional ball, one gets that

$$\begin{aligned} \frac{V_d(1)}{V_{d-1}(1)}= &   \frac{\pi ^{d/2}/\Gamma (d/2 + 1)}{\pi ^{(d-1)/2}/\Gamma ((d-1)/2 + 1)}\nonumber \\= &   \pi ^{1/2} \frac{\Gamma (d/2 + 1/2)}{\Gamma (d/2 + 1)} > \pi ^{1/2} \frac{\sqrt{2}}{\sqrt{d+\frac{1}{2}}} \ge \sqrt{\frac{\pi }{d}}, \end{aligned}$$

where we have used the inequality by Kershaw [26]

$$ \forall s\in (0,1),~ \forall x\ge 1:~~~ \left( x+\frac{s}{2} \right) ^{s-1} <~ \frac{\Gamma (x+s)}{\Gamma (x+1)} $$

for x=d/2, which is at least 1 for d≥2, and for s=1/2, which gives

$$ \left( \frac{d+1/2}{2} \right) ^{-1/2} ~<~ \frac{\Gamma (d/2+1/2)}{\Gamma (d/2+1)}. $$

(For d=1 we can just check that $\Gamma (1) = 1 > \frac{\sqrt{\pi }}{2}=\Gamma (3/2)$.) The result follows. □

**Fig. 6:**
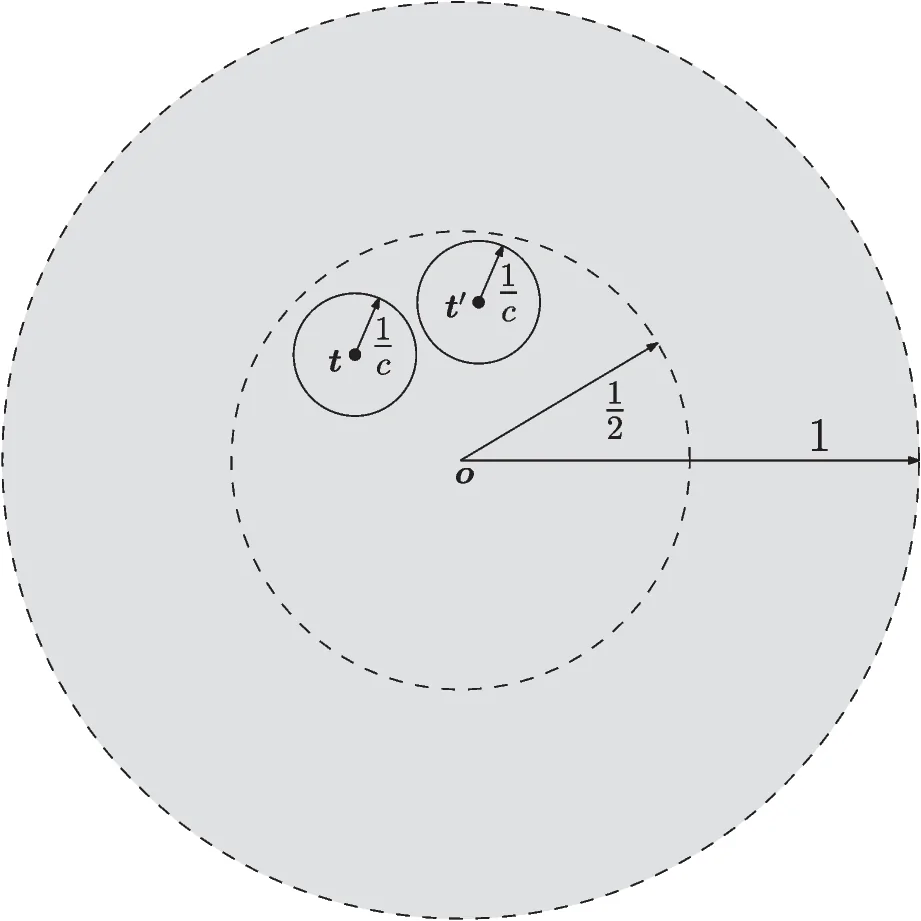
Parts in the domains for $\lambda _{\boldsymbol{t}}$ and $\lambda _{\boldsymbol{t}'}$ for two targets when d=2

**Fig. 7:**
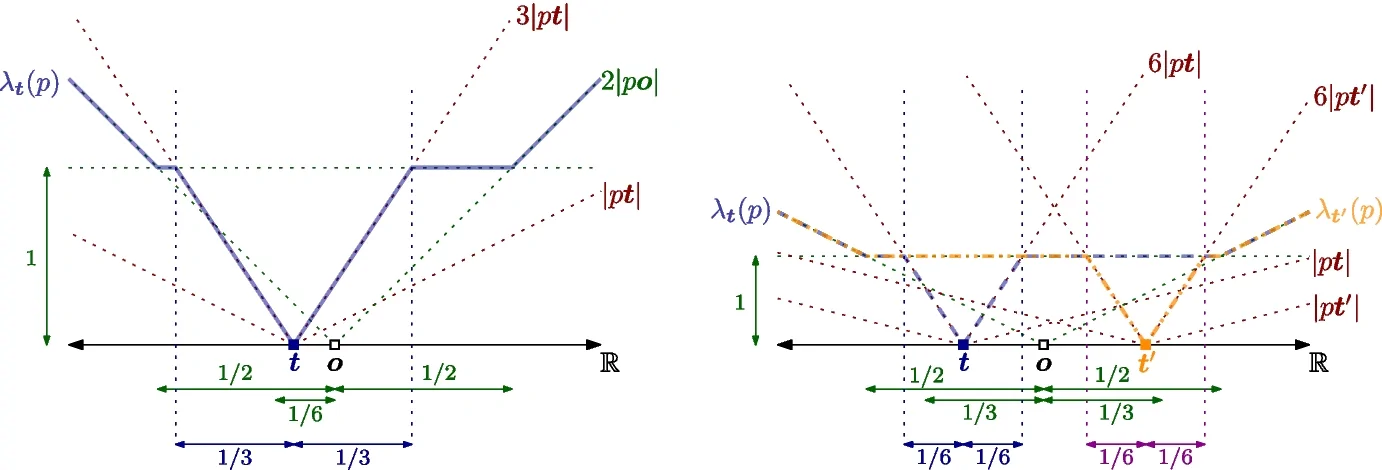
Examples of functions $\lambda _{\boldsymbol{t}}$ for d=1. Note that the axes have different scales. Left: example for c=3; the target has to be at distance at most 1/6 from the origin. Right: two functions for c=6; the target has to be at distance at most 1/3 from the origin

Next, fix a value c≥2. For each $\boldsymbol{t}\in B(\boldsymbol{o},1/2 - 1/c)$, let $\lambda _{\boldsymbol{t}}$ be the function defined as follows; see Figs. 6 and 7 for intuition.

2 $$\begin{aligned} \lambda _{\boldsymbol{t}} (p) = \left\{ \begin{array}{ll} c \cdot |p\boldsymbol{t}|, & \text { if } p\in B(\boldsymbol{t}, 1/c) ,\\ 1, & \text { if } p\in B(\boldsymbol{o},1/2)\setminus B(\boldsymbol{t}, 1/c) ,\\ 2\cdot |p\boldsymbol{o}|, & \text { if } p\notin B(\boldsymbol{o},1/2) . \end{array}\right. \end{aligned}$$

### Lemma 11

Assume that c≥2 and $|\boldsymbol{o}\boldsymbol{t}| \le \tfrac{1}{2}- \tfrac{1}{c}$. Then the function $\lambda _{\boldsymbol{t}}$ is a *c*-prediction for the target $\boldsymbol{t}$. Moreover, for any two distinct $\boldsymbol{t},\boldsymbol{t}'$ satisfying the hypothesis, the functions $\lambda _{\boldsymbol{t}}$ and $\lambda _{\boldsymbol{t}'}$ agree on all points outside $B(\boldsymbol{t}, 1/c)\cup B(\boldsymbol{t}', 1/c)$.

### Proof

Consider any fixed $\boldsymbol{t}\in B(\boldsymbol{o},1/2- 1/c)$. We have to show that $\lambda _{\boldsymbol{t}}$ satisfies

$$ \forall p\in \mathbb R^d: ~~~ |p\boldsymbol{t}| ~\le ~ \lambda _{\boldsymbol{t}}(p) ~\le ~ c \cdot |p\boldsymbol{t}|. $$

We do this by considering points in each part of the domain used to define $\lambda _{\boldsymbol{t}}$.

The first part is easy as $|p\boldsymbol{t}| < \lambda _{\boldsymbol{t}}(p) = c \cdot |p\boldsymbol{t}|$ for every $p\in B(\boldsymbol{t}, 1/c)$. For the second part we note that:

$$\begin{aligned} \forall p\in B(\boldsymbol{o},1/2)\setminus B(\boldsymbol{t}, 1/c)&: ~~~ |p\boldsymbol{t}| ~\le ~ |p\boldsymbol{o}| + |\boldsymbol{o}\boldsymbol{t}| ~\le ~ \frac{1}{2} + \frac{1}{2} ~=~ 1 ~=~ \lambda _{\boldsymbol{t}}(p).\\ \forall p\in B(\boldsymbol{o},1/2)\setminus B(\boldsymbol{t}, 1/c)&: ~~~ \lambda _{\boldsymbol{t}}(p) ~=~ 1 ~=~ c\cdot \frac{1}{c} ~\le ~ c \cdot |p\boldsymbol{t}|. \end{aligned}$$

For the last part we first note:

$$\begin{aligned} \forall p\notin B(\boldsymbol{o},1/2)&: ~~~ |p\boldsymbol{t}| ~\le ~ |p\boldsymbol{o}| + |\boldsymbol{o}\boldsymbol{t}| ~\le ~ |p\boldsymbol{o}| + \frac{1}{2} ~\le ~ 2\cdot |p\boldsymbol{o}| ~=~ \lambda _{\boldsymbol{t}}(p). \end{aligned}$$

For each $p\notin B(\boldsymbol{o},1/2)$, let *q* be the point where the segment $p\boldsymbol{t}$ crosses the boundary of $B(\boldsymbol{o},1/2)$. Thus, $|q\boldsymbol{o}|=1/2$ and, since $B(\boldsymbol{t},1/c)\subseteq B(\boldsymbol{o},1/2)$, we also have $|q\boldsymbol{t}|\ge 1/c$. We then have, using that c≥2,

$$\begin{aligned} \forall p\notin B(\boldsymbol{o},1/2) : ~~~ \lambda _{\boldsymbol{t}}(p) ~&=~ 2\cdot |p\boldsymbol{o}| ~\le ~ 2\cdot \big (|pq|+|q\boldsymbol{o}|\big ) ~=~ 2\cdot |pq| + 1 \\ ~&\le ~ 2\cdot |pq| + c\cdot |q\boldsymbol{t}| ~\le ~ c\cdot \big (|pq|+|q\boldsymbol{t}|\big ) ~=~ c\cdot |p\boldsymbol{t}|. \end{aligned}$$

This covers all cases for the domain of $\lambda _{\boldsymbol{t}}$ and concludes the proof that $\lambda _{\boldsymbol{t}}$ is a *c*-prediction.

From the definition of $\lambda _{\boldsymbol{t}}$ it is clear that $\lambda _{\boldsymbol{t}}(p)=\lambda _{\boldsymbol{t}'}(p)$ for all $p\notin B(\boldsymbol{t},1/c)\cup B(\boldsymbol{t}',1/c)$ because its value is independent of $\boldsymbol{t}$ and $\boldsymbol{t}'$. □

### Theorem 12

Assume that c>2. There is family of *c*-predictions for targets in $B(\boldsymbol{o},1/2-1/c)$ such that any search path in $\mathbb R^d$ that uses *c*-predictions to find the target has length at least $\left( \frac{c-2}{2}\right) ^d \cdot \frac{1}{c} \cdot \min \{\sqrt{\pi /d},1\}$.

### Proof

For each $\boldsymbol{t}\in B(\boldsymbol{o},1/2 - 1/c)$, consider the function $\lambda _{\boldsymbol{t}}$ defined in (2). Lemma 11 shows that the function $\lambda _{\boldsymbol{t}}$ is a *c*-prediction for each $\boldsymbol{t}\in B(\boldsymbol{o},1/2 - 1/c)$. Consider the set of prediction functions $\Lambda = \{ \lambda _{\boldsymbol{t}}\mid \boldsymbol{t}\in B(\boldsymbol{o},1/2 - 1/c)\}$. Let $\mathbb B=\{ B(\boldsymbol{t},1/c)\mid \boldsymbol{t}\in B(\boldsymbol{o},1/2 - 1/c)\}$. Note that to distinguish between two functions $\lambda _{\boldsymbol{t}},\lambda _{\boldsymbol{t}'}\in \Lambda$ we have to evaluate them at some point of $B(\boldsymbol{t},1/c)\cup B(\boldsymbol{t}',1/c)$.

Consider any search strategy when the *c*-prediction function is selected from Λ by an *adversary*. The search strategy may exploit that the *c*-prediction is from Λ and, in particular, it may exploit that the target $\boldsymbol{t}$ is a point of $B(\boldsymbol{o},1/2 - 1/c)$, selected by the adversary. We claim that the search path has to visit all the balls of B. Indeed, while there are two distinct points $\boldsymbol{t},\boldsymbol{t}' \in B(\boldsymbol{o},1/2 - 1/c)$ such that the balls $B(\boldsymbol{t},1/c)$ and $B(\boldsymbol{t}',1/c)$ are not visited by the search path, we have $\lambda _{\boldsymbol{t}}(p)=\lambda _{\boldsymbol{t}'}(p)$ for all points *p* along the path, and therefore the information collected cannot discern whether the target is $\boldsymbol{t}$ or $\boldsymbol{t}'$. Thus, in the worst case, the search path has to visit all the balls of B but one, to identify which point of $B(\boldsymbol{o},1/2 - 1/c)$ is the target, and then still may have to move to that last ball. That is, we may assume that in the worst case the adversary chooses $\lambda _{\boldsymbol{t}}\in \Lambda$ such that $B(\boldsymbol{t},1/c)$ is the *last* ball of B visited by the search strategy. After the searcher deduces at which point of $B(\boldsymbol{o},1/2 - 1/c)$ the target is, they still have to move to the target.

We conclude that the search path has to visit all balls of B, and for the last ball it still has to travel 1/*c* to the center. Lemma 10 (with δ=1/c) implies that the search path has length at least

$$\begin{aligned} \frac{1}{c} + \left( \Big (\frac{c}{2}-1 \Big )^d - 1\right) \cdot \frac{1}{c}\cdot \sqrt{\frac{\pi }{d}} ~&\ge ~ \frac{1}{c} + \left( \Big (\frac{c-2}{2}\Big )^d - 1\right) \cdot \frac{1}{c} \cdot \min \{\sqrt{\pi /d},1\} \\&\ge ~ \left( \frac{c-2}{2}\right) ^d \cdot \frac{1}{c} \cdot \min \{\sqrt{\pi /d},1\}. \end{aligned}$$

□

For the *competitive ratio* we have to compare the length of the search path to the distance to the target, which in the construction is at most 1/2-1/c. We then obtain the following bound, which is non-trivial when d≥2 and c>2.

### Corollary 13

Consider the search with predictions problem in $\mathbb R^d$ with prediction factor c>2. Any search strategy to reach the target has competitive ratio at least $\left( \frac{c-2}{2}\right) ^{d-1}\cdot \min \{\sqrt{\pi /d},1\}$.

### Proof

Consider the construction of Theorem 12. Since the search path has length at least $\left( \frac{c-2}{2}\right) ^d \cdot \frac{1}{c} \cdot \min \{\sqrt{\pi /d},1\}$ and the target is $\boldsymbol{t}\in B(\boldsymbol{o},1/2-1/c)$, the competitive ratio is at least

$$\begin{aligned} \frac{\left( \frac{c-2}{2}\right) ^d \cdot \frac{1}{c} \cdot \min \{\sqrt{\pi /d},1\}}{|\boldsymbol{o}\boldsymbol{t}|}&\ge ~ \frac{\left( \frac{c-2}{2}\right) ^d \cdot \frac{1}{c} \cdot \min \{\sqrt{\pi /d},1\}}{1/2-1/c} \\&=~ \frac{\left( \frac{c-2}{2}\right) ^d \cdot \frac{1}{c} \cdot \min \{\sqrt{\pi /d},1\}}{\frac{c-2}{2c}}\\&=~ \left( \frac{c-2}{2}\right) ^{d-1} \cdot \min \{\sqrt{\pi /d},1\}. \end{aligned}$$

□

To get a bound that is easier to grasp, let us assume that c≥4. In this case (c-2)/2≥c/4 and we get the following bound.

### Corollary 14

Consider the search with predictions problem in $\mathbb R^d$ with prediction factor c≥4. Any search strategy to reach the target has competitive ratio at least $(c/4)^{d-1}\cdot \min \{\sqrt{\pi /d},1\}$.

The lower bounds still hold in the case where the target has integral coordinates or is detected when we are within a given distance, with a slightly worse constant in the basis of the exponential function. For this, we can just scale the input such that the balls in the construction are large enough to contain points with integer coordinates. Then, we still have to visit all balls in order to detect which one has the target.

A similar lower bound holds for randomized search strategies. In our construction, the searcher has to visit some balls in the family B of balls of Lemma 10 (with δ=1/c), and they can deduce something only when visiting the ball whose center is the target. In the worst-case (or adversary) model, the target is going to be the center of the last ball of B that is visited. In a randomized setting, we select the target *uniformly* at random among all the possible ones. This means that $\boldsymbol{t}$ is selected uniformly at random among the points of $B(\boldsymbol{o},1/2-1/c)$. The intuition is that, when the search path has touched half of the balls of B (in a measure theoretic sense), with probability 1/2 the ball $B(\boldsymbol{t},1/c)$ has been touched. We next provide the details.

### Lemma 15

Let π be a path in $\mathbb R^d$ and let *p* be a point selected uniformly at random in $B(\boldsymbol{o},1/2-\delta )$, where $\delta \le 1/2$. If π touches B(p,δ) with probability at least 1/2, then π has length at least $\left( \frac{1}{2}\cdot \big (\frac{1}{2\delta }-1 \big )^d - 1\right) \cdot \delta \cdot \sqrt{\frac{\pi }{d}}$.

### Proof

Let *L* be the length of the path π that touches B(p,δ) with probability at least 1/2, when *p* is selected uniformly at random from $B(\boldsymbol{o},1/2-\delta )$. The argument used in the proof of Lemma 10 implies the volume inequality

$$ {{\,\textrm{vol}\,}}\big ( \pi \oplus B(\boldsymbol{o},\delta ) \big ) ~\ge ~ \frac{1}{2}\cdot {{\,\textrm{vol}\,}}\big ( B(\boldsymbol{o},1/2-\delta )\big ). $$

Using the observations and the notation in the proof of Lemma 10, we have

$$ {{\,\textrm{vol}\,}}\big ( \pi \oplus B(\boldsymbol{o},\delta ) \big ) ~\ge ~ \frac{1}{2}\cdot {{\,\textrm{vol}\,}}\big ( B(\boldsymbol{o},1/2-\delta )\big ) ~=~ \frac{1}{2}\cdot \left( \frac{1}{2}-\delta \right) ^d\cdot V_d(1). $$

and

$$ {{\,\textrm{vol}\,}}\big ( \pi \oplus B(\boldsymbol{o},\delta ) \big ) ~\le ~ V_d(\delta ) + L \cdot V_{d-1}(\delta ) ~=~ \delta ^d \cdot V_d(1) + L \cdot \delta ^{d-1} \cdot V_{d-1}(1). $$

Therefore

$$\begin{aligned} L ~&\ge ~ \frac{\frac{1}{2}\cdot \left( \frac{1}{2}-\delta \right) ^d\cdot V_d(1) - \delta ^d \cdot V_d(1)}{\delta ^{d-1} \cdot V_{d-1}(1)} ~=~ \left( \frac{1}{2}\cdot \Big (\frac{1}{2\delta }-1 \Big )^d - 1\right) \cdot \delta \cdot \frac{V_d(1)}{V_{d-1}(1)} \\ ~&\ge ~ \left( \frac{1}{2}\cdot \Big (\frac{1}{2\delta }-1 \Big )^d - 1\right) \cdot \delta \cdot \sqrt{\frac{\pi }{d}}. \end{aligned}$$

□

### Theorem 16

Assume that c>2. There is family Λ of *c*-predictions for targets in $B(\boldsymbol{o},1/2-1/c)$ with the following property. If a prediction is selected uniformly at random from Λ, then any search strategy in $\mathbb R^d$ that uses *c*-predictions to find the target has, with probability at least 1/2, length at least $\frac{1}{2}\cdot \left( \frac{c-2}{2}\right) ^d \cdot \frac{1}{c} \cdot \min \{\sqrt{\pi /d},1\}$.

### Proof

We adapt the proof of Theorem 12. Consider the family $\Lambda = \{ \lambda _{\boldsymbol{t}}\mid \boldsymbol{t}\in B(\boldsymbol{o},1/2 - 1/c)\}$ of *c*-prediction functions and assume that $\lambda _{\boldsymbol{t}}$ is selected from Λ uniformly at random (by selecting $\boldsymbol{t}\in B(\boldsymbol{o},1/2 - 1/c)$ uniformly at random).

Let π be the search path followed by the search strategy. Unless π touches $B(\boldsymbol{t},1/c.)$, it obtains no information about the target. After the searcher hits $B(\boldsymbol{t},1/c)$ for the first time, it still has to move to the target, which costs an additional 1/*c*.

Using Lemma 15 with δ=1/c, we conclude that, with probability at least 1/2, the search path has length at least

$$\begin{aligned} \frac{1}{c} \!+\! \left( \frac{1}{2}\cdot \Big (\frac{c}{2} \!-\! 1 \Big )^d \!-\! 1\right) \cdot \frac{1}{c}\cdot \sqrt{\frac{\pi }{d}} ~&\!\ge \! \frac{1}{c} \!+\! \left( \frac{1}{2}\cdot \Big (\frac{c-2}{2}\Big )^d \!-\! 1\right) \cdot \frac{1}{c} \cdot \min \{\sqrt{\pi /d},1\} \\&\ge ~ \frac{1}{2}\cdot \left( \frac{c-2}{2}\right) ^d \cdot \frac{1}{c} \cdot \min \{\sqrt{\pi /d},1\}. \end{aligned}$$

□

It follows from Theorem 16 that the expected length of the path of any search strategy, randomized or not, is at least $\frac{1}{4}\cdot \left( \frac{c-2}{2}\right) ^d \cdot \frac{1}{c} \cdot \min \{\sqrt{\pi /d},1\}$. This is 1/4 smaller than the bound in Theorem 12 for worst-case scenarios. We conclude that the lower bounds in Corollaries 13 and 14 hold for randomized strategies, but the lower bound for the expected competitive ratio is worse by a factor of 1/4.

## Conclusions and Research Directions

We have introduced and studied the problem of searching for a target in $\mathbb R^d$ under a predictions model in which the searcher is given, at each position they visit, an approximate distance to the target. We presented strategies with competitive ratio of $O(c^d)$, where *c* is the prediction factor, which may be unknown. We also showed a lower bound of $(c/4)^{d-1} \cdot \min \{\sqrt{\pi /d},1\}$ for the competitive ratio of any strategy under this model, assuming that c≥4, even when the prediction factor *c* is known.

Let us discuss some of the weaknesses of our techniques to obtain the bounds. Our search strategies use the estimate for the distance to the target only at a finite number of points through the search, but the model permits obtaining the estimate continuously. Thus, we are not exploiting all the properties of the model. Also, our search strategies conduct a search through a sequence of points until it hits a point where the estimate for the distance decreases by 1/2. However, it may be better to stop the iteration over the sequence of points if early on we hit a point where, say, the estimate decreases by 3/4. As for the lower bound, it may be that an adversary that adapts to the search strategy may provide a better lower bound. Our current lower bound largely ignores the search path and just keeps a constant estimate for the distance through most of the search.

It will be interesting to revisit linear search and search in concurrent rays under this paradigm. Here, one can of course use the well-known linear search strategies without predictions, but we expect that predictions throughout the whole search will lead to a better competitive ratio. Previous works [10, 22, 30] where a prediction is only given at the start, already provided a starting ground for this and showed that better search strategies are possible.

The case of c=1+ε for sufficiently small ε>0 seems also interesting, because in this case the spherical shells are very thin, and perhaps one can provide better bounds.

A natural question is how to bring the upper and lower bounds closer. Here, it will be interesting to examine whether randomization can improve the competitive ratios of our strategies, similarly to the case of linear and multiple ray search [17, 24, 25].

Considering the problem in other metric spaces, such as graphs, and searching for more complex targets, such as convex subsets or *k*-flats, seems also a fruitful line of research. For example, does searching for a *k*-flat in $\mathbb R^d$ with predictions behaves like searching for a point in $\mathbb R^d$ or like a point in $\mathbb R^{d-k}$?

One can also study whether weaker assumptions on the prediction function suffice to achieve constant competitive ratio. For example, if the prediction $\lambda (\cdot )$ satisfies $|p\boldsymbol{t}|\le \lambda (p)\le 2\cdot |p\boldsymbol{t}|+|p\boldsymbol{t}|^2$ for all $p\in \mathbb R^d$, can one still achieve constant competitive ratio? In the spirit of [5], can we afford having completely wrong predictions in some region of $\mathbb R^d$ with non-zero Lebesgue measure, if the region does not contain the target?

Finally, another future line of research is studying the classical geometric variants of pursuit-evasion problems with mobile targets [1, 17] under our predictions model.

## Data Availability

No datasets were generated or analysed during the current study.
